# Control Work

**Published:** 1898-01

**Authors:** 


### CONTROL WORK.

---

*National.*—Much interest has been aroused by the aspect of bureau affairs at Kansas City, Mo., where some dissensions between the inspectors of the Bureau of Animal Industry relative to the condemnation of certain animals in the abattoirs there, culminating in charges preferred against one of the packers on the ground of attempting to bribe one of the inspectors. The matter has been taken to the Federal grand-jury, and attorneys for the packer have entered a demurrer on the grounds that the law establishing inspection is unconstitutional, and consequently said inspectors are not United States officers and that the inspection is unlawful. This is a similar ground to that which was taken during the period when contagious pleuro-pneumonia was being investigated, which was finally settled in favor of the Government.

*Minnesota.*—The State Board of Health, after a conference with the Secretary and Director of the Agricultural Society, prescribed the conditions under which swine might come to the Minnesota State Fair last fall ; the swine were inspected upon arrival and daily during the fair week, because of the prevalence of hog-cholera in the State. No disease appeared among the swine upon the fair-grounds, and it is hoped that the exhibit this year will not result in any increased spread of hog-cholera.

*Pennsylvania.*—The alarming condition of the State Treasury resulted in the announcement, at the close of November, that no further appropriations would be made to the Live-stock Sanitary Board, and its work stopped. State Veterinarian Pearson, fully realizing the dangers of this condition, and how serious a matter to the live-stock interests of the State, and the false economy such a move would prove, asked at once for a meeting of the board, and presented the situation that would follow, referring to the progress of protective work among the live-stock owners of the State, who were joining in every effort to aid the authorities in freeing our State from the disastrous and dangerous diseases to man and our commercial interests, and succeeded in having an appropriation made for the next three months' work. He stated that 406 herds had been examined the past six months, numbering 5368 cattle, of which number 658 were found diseased and destroyed. That the percentage of diseased ones had fallen from 20.2 per cent. to 12.2 per cent., showing that the worst centres of disease had been discovered and removed.

*New York.* Work at the St. Lawrence State Hospital showed that out of a herd of 114, some 82 were found tuberculous. The State Board of Health has examined some 948 others, a number of which were found diseased, and destroyed.

